# The Triple Challenge of Intergenerational Sandwiching and Labor-Force Participation: Consequences for the Psychological Well-Being of Older Europeans

**DOI:** 10.21203/rs.3.rs-10382527/v1

**Published:** 2026-09-02

**Authors:** Marco Albertini, Noah Lewin-Epstein, Merril Stilverstein, Aviad Tur-Sinai

**Affiliations:** University of Bologna; Tel Aviv University; Syracuse University; University of Haifa

## Abstract

Individuals sandwiched between generations while being in paid work, face a triple challenge and may experience significant negative psychological effects associated with these three competing time demands. This study adds to the literature by focusing on the “mature” sandwich generation in Europe and examining the consequences of transitioning into social sandwiching—simultaneously providing support to older parents and adult children – on individuals’ psychological well-being. Attention is given to gender differences in the context of paid work, retirement, and non-employment. Using data from the Survey of Health, Ageing and Retirement in Europe covering 2004–2022, we estimate symmetric fixed-effects linear regression models, separately by gender and employment status, to assess within-person changes in psychological well-being following a transition into social sandwiching. Psychological well-being is measured by two complementary indicators: the number of reported depressive symptoms (Euro-D) and the quality of life (CASP12). Transitioning into social sandwiching was associated with an increased number of depressive symptoms and reduced levels of subjective well-being among women in paid work. No significant effects were observed among women outside the labor force. No negative effects were found among employed men; however, retired men experienced a decline in subjective well-being.

Findings underscore the psychological costs of combining paid work with multigenerational caregiving, particularly for women. The results highlight the need for flexible labor policies that mitigate work–family strain by specifically addressing the needs of an aging workforce facing an increasing likelihood of social sandwiching.

## Introduction

Increased life expectancy and the resulting increase in three- and even four-generation families make it particularly important to improve our understanding of the challenges faced by those providing support to multiple adult generations and the implications for their well-being. With delayed parenthood and increased longevity, middle-aged individuals often find themselves demographically sandwiched between aging parents and adult offspring (Železná 2018). Estimates suggest that one-third of individuals fifty years of age are members of this mature sandwich generation (Friedman et al. 2017; Herlofson and Brandt 2020). The current study contributes to the body of knowledge on social sandwiching by focusing on adults at an age when their parents are aging and typically require assistance while their adult children may be facing economic and childcare challenges that parents can help meet. In our study, we distinguish between social sandwiching—providing support up and down the generational ladder—and demographic sandwiching, the structural position of being in a middle generation.

Managing conflicting demands on time-resource allocation is one of the main challenges that the mature sandwich generation—middle-aged individuals who have surviving parents, adult children, and grandchildren (Albertini et al. 2022)—faces. Although the well-being of the sandwich generation has been the topic of many studies, the adverse contribution of social sandwiching to well-being among the older working-age population has been inadequately studied despite the growth of this demographic. Many sandwiched individuals who support members of younger and older generations are also employed in the labor market and are likely to sustain additional stress and adverse effects on well-being as a result.

Research on social sandwiching—providing support to both younger and older generations—in conjunction with employment has focused mostly on the difficulties faced by women with dependent children (e.g., Ansari-Thomas 2024). Much less attention has been devoted to late middle-aged women and men whose parents and adult children are both in need of assistance. Previous research has reported that social sandwiching is detrimental to the psychological well-being of older individuals, particularly older women (Albertini et al. 2024). Meeting the demands of family members of other generations while remaining in the workforce is particularly challenging to members of this older age group (Kridahl and Silverstein 2020). In a recent scoping review of fifteen empirical studies, Hodgdon et al. (2023) concluded that sandwiched caregivers occupied a unique position that attests in lower levels of psychological well-being than those among other types of family caregivers and non-caregivers. Although most studies on the topic document the deleterious effect of being socially sandwiched, it should be noted that several (e.g., White-Means 2017) found no difference between sandwiched and non-sandwiched caregivers with respect to emotional strain or burden.

A critical yet underexplored aspect of social sandwiching is the dynamic nature of this family position, which is entered and exited over the family lifecycle. While much of the existing research focuses on static comparisons between sandwiched and non-sandwiched individuals, we gain a better understanding of the ways these temporally changing responsibilities impact individuals by analyzing transitions into and out of sandwiching. Given that paid work exacerbates the time demands placed on caregivers, we consider the moderating role of labor-market participation in amplifying the impact of transitioning into social sandwiching on older workers’ psychological well-being. By shifting the focus to sandwiching transitions, we aim to avoid issues of self-selection and the role of time-invariant individual characteristics and to provide a more nuanced person-focused perspective on the evolving pressures experienced by the mature sandwich generation in their working and family lives. Thus, a distinctive contribution of this study is its focus on *within-person transitions* into and out of social sandwiching. By adopting a longitudinal fixed-effects approach, we capture how changes in caregiving responsibilities alter individual well-being net of stable personal and contextual characteristics. This dynamic perspective provides a stronger basis for identifying and interpreting the psychological costs of becoming sandwiched than that of studies that contrast sandwiched and non-sandwiched individuals at one point in time. Furthermore, by examining both men and women in late midlife, our analysis broadens the scope of research that has disproportionately centered on younger women, thereby illuminating how gendered caregiving and work patterns shape well-being at older working ages as well.

### Theoretical Frameworks and Previous Research

As suggested above, socially sandwiched individuals who continue to work for pay face a triple challenge in managing the combined time demands on their working and family lives. The harmful effects of time-demand overload on psychological well-being may be explained by three social mechanisms that operate at the individual level and are illuminated within different theoretical frameworks: role theory (role overload and conflict), resource-depletion theory (stress associated with time poverty), and control theory (perceived loss of control due to excessive demands). In the following sections, we briefly review the relevance of these frameworks to our investigation and then formulate our research goals and hypotheses.

### Micro-Level Stress Mechanisms

Several theoretical constructs are employed in the literature to describe the implications of social sandwiching for the psychological well-being of sandwiched individuals. Some studies (Feng et al. 2023; Hammen 2005) invoke the psychological construct of “stress” to explain psychological outcomes related to role overload. Stress arises in situations of high and conflicting demands on one’s resources and difficulty in prioritizing the allocation of these resources. Other studies turn to “resource depletion” theory, suggesting that the labor demands of work and responsibilities to family overwhelm available time and energy resources (Halinski et al. 2018; Duxbury et al. 2022). In a similar vein, some authors turn to the concept of “role overload” and its challenges to allocating limited time resources to meet multiple social obligations (Ford et al. 2007). The three theories—stress, role conflict, and resource depletion—are closely related and have found credence in time-budget studies that document adverse psychological effects of time poverty (Williams et al. 2016).

An additional body of research focuses on the perception of loss of control and the strain that results when one attempts to meet multiple others’ needs. Karasek, for example, (Karasek 1979; Karasek and Theorell 1990) noted that a low sense of control on the job exacerbates the strain associated with multiple demands. To facilitate employee productivity, well-being, and retention, organizational policies often give employees in such situations more control over how they allocate their time (Halinski et al. 2018; Turgeman-Lupo et al. 2020). This perspective aligns with broader research on the work–life balance that emphasizes the role of employer policies in shaping the ability of socially sandwiched individuals to sustain their employment while fulfilling caregiving duties.

Research on the well-being of multigenerational caregivers who are also employed calls attention to their diminished levels of psychological well-being and health, higher levels of stress (Boyczuk and Fletcher 2016; Halinski et al. 2018; Remennick 2001; Turgeman-Lupo 2020), and degraded job satisfaction (Sahibzada et al. 2005). Studies that provide detailed measures of perceived work–family stress find that sandwiched caregivers experience two-way negative spillovers between family and work contexts, giving rise to depressive symptoms (e.g., Depasquale et al. 2016; Hodgdon and Wong 2019; Neal and Hammer 2009; Turgeman-Lupo et al. 2020). Evidence of additive stress of family demands among workers emerges from research showing higher levels of work–family role overload and perceived stress among socially sandwiched workers than among workers who provide eldercare or childcare only (Ford et al. 2007). Irrespective of the theoretical constructs employed, all these theories emphasize how facing demanding responsibilities across several domains results in deleterious outcomes for mental as well as physical health (Grünwald et al. 2021; Liu et al. 2020; Wiemers and Bianchi 2015).

### Study Contribution and Hypotheses

We postulate that stress caused by multiple family demands is most consequential for negative outcomes when accompanied by work demands. In our application, we expect the transition into social sandwiching to impose significant psychic costs when added to work obligations. This is best captured by examining transitions into and out of sandwiching with panel data in conjunction with work status. We treat sandwiching as a dynamic process that one may enter (starting to support a parent while still supporting a child, for example) or leave (ending support for a child while experiencing the death of a supported parent, *inter alia*).

In the present study, we wish to enhance understanding of the relationship among social sandwiching, paid work, and well-being in several ways. First, most studies on the outcomes of intergenerational sandwiching rely on cross-sectional comparisons between sandwiched and non-sandwiched individuals, leaving open the possibility that dispositional factors (such as poor mental health or pre-selection of individuals out of the labor force) bias the results. By focusing on changes in psychological well-being following transitions into and out of social sandwiching in the context of different employment statuses, the present study largely overcomes this limitation.

Second, the age group of sandwiched individuals on which we focus is older than that in most previous studies—a unique population of late working-age individuals who are most likely to simultaneously have parents reaching advanced old age and needing considerable care and children in young adulthood who may have children of their own who require support and care.

Third, studies on the impact of social sandwiching and work are far less common among men than among women. Given known gender differences in care provision and participation in the paid labor market, we investigate the relationship among sandwiching transitions, employment status, and well-being separately for women as contrasted with men. The gender focus is relevant due to gender differences in family care and characteristics of the work environment. Caregiving tends to be delivered more intensively by women than by men, manifested in more emotionally and time-demanding activities (such as personal and emotional care), as against less demanding support provided by men (such as help with paperwork, errands, or transportation)—uniquely amplifying the work–family stress that women caregivers who engage in paid work experience. Our focus on both gender groups allows us to contrast distinct vulnerabilities because women workers are more likely than men to invest heavily in family roles, namely, to undertake the so-called “second shift” (Hochschild 2012).

Disadvantages that women in the labor force face may also explain the association between multigenerational caregiving and adverse outcomes among working women (Henle et al. 2020). Women tend to hold less flexible jobs than those held by men, possibly rendering them unable to modify their work schedule to care for parents, children, or grandchildren (Cravey and Mitra 2011; Carrino et al. 2023) and creating work–family anxiety (Azami et al. 2018).

Synthesizing the literature on stress related to work–family conflict, our first hypothesis proposes that a transition into social sandwiching among paid workers will produce a decline in psychological well-being and that this effect will be weaker or non-existent among those not in paid work. Given known gender differences in work arrangements and care provision, our second hypothesis proposes that this deleterious effect will be more pronounced among women than among men.

## Data and Methods

### Sample

We base our analysis on data from the Survey of Health, Ageing and Retirement in Europe (SHARE). SHARE is a longitudinal, multidisciplinary cross-national survey representative of the non-institutionalized population aged 50 + in several European countries. Our respondents came from an array of countries, including Austria, Germany, Sweden, the Netherlands, Spain, Italy, France, Denmark, Greece, Switzerland, Belgium, Israel, the Czech Republic, Poland, Luxembourg, Hungary, Portugal, Slovenia, Estonia, Croatia, Lithuania, Bulgaria, Cyprus, Finland, Latvia, Malta, Romania, and Slovakia.

We utilized data from Release 9.0.0 of seven regular waves of the survey (that is, excluding retrospective waves): Waves 1, 2, 4, 5, 6, 8, and 9, corresponding to years 2004/05, 2006/07, 2011, 2013, 2015, 2019/2020, and 2021/22, respectively. The analytical sample consists of observations of all individuals aged 50–65 who participated in at least two regular waves of SHARE, experienced no change in employment status, and had no missing information on the variables utilized in the multivariate regression models. By selecting individuals who participated in more than one wave of the survey, we were able to estimate the association between transitioning into/out of social sandwiching and change in psychological well-being. (See Albertini et al. 2024 for use of a similar approach.) We also omitted 23% of person-observations that found a change in employment status in order to avoid challenging the statistical model by interacting time-varying factors. (For possible consequences of this selection, see the Discussion.) With these selection criteria, the analytic sample consisted of 46,773 individuals who contributed 121,109 observations to the analysis.

### Variables

Psychological well-being was represented by two scales, one measuring psychological distress (PD) and the other reflecting subjective well-being (SWB). Psychological distress was measured by the EURO-D scale, a well-used and validated measure of depressive symptoms among older adults (Castro-Costa et al. 2008; Prince et al. 1999). SWB was captured by CASP12 (Hyde et al. 2015), which measures the degree of individual satisfaction and fulfillment in four conceptual domains of psychological health: control, autonomy, self-realization, and pleasure (for elaboration on the items used to construct the measures, see Mehrbrodt et al. 2021). Both scales have been shown adequately reliable and valid in regard to SHARE data (Courtin, et al. 2015; Oliver et al. 2021).

To detect social sandwiching, we constructed a time-varying dichotomous variable that indicates whether the respondent simultaneously provided support to adult children and parents and/or parents-in-law in the twelve months preceding the interview. The following types of support were considered: personal care, support with household chores, paperwork, financial support, co-residing, and, among adult children, looking after grandchildren. (See Albertini et al. 2022 for justification of this operationalization of social sandwiching.) The transition into social sandwiching may occur from one of two distinct previous states: providing support to a member of only one generation or providing support to a member of neither generation.

Employment status was considered at each wave and divided into three mutually exclusive categories: “in paid work,” “retired,” and “other not in paid work.” The last group is a residual category of those not working but not retired, a designation that includes the unemployed, those who never worked, homemakers, and the disabled. Full-time and part-time work are treated interchangeably; robustness checks considering only full-time work yielded comparable results.

The regression model controlled for the following time-varying characteristics: age, physical health (number of diagnosed chronic diseases and activity limitations using the Global Activity Limitation Indicator [hereinafter: GALI]), and various sociodemographic characteristics including the number of grandchildren, having a co-residing partner, and the partner’s employment status.

### Analytic Strategy

In accordance with our focus on within-individual transitions in the presence/absence of social sandwiching and corresponding variation in mental health, we estimated a symmetric fixed-effects linear regression. Fixed-effects models are well suited to identifying causal influences because time-varying variables are evaluated relative to the respondent’s overall average on each of these variables. As a result, time-invariant characteristics such as gender, country of residence, level of education, and all other unchanging factors are rendered inconsequential.

Given known gender differences in work and family roles, we estimated models separately for male and female respondents. Due to the limited statistical power of the data, we refrain from estimating the effects of employment as a time-varying variable, and we considered only those individuals whose employment status did not change across observations. Therefore, the regression models are fitted to six separate subsamples of respondents, each subsample corresponding to the combination of gender group (male/female) and employment status (in paid work/retired/other, not in paid work).

Distributions of key variables and other descriptive statistics for male and female observations are found in Table 1. The average age is late 50s for men and women (59 and 58, respectively). A majority of men (60%) and a plurality of women (49%) were in paid work across all observations and about one-quarter of each gender was retired (25% and 24%, respectively).

Social sandwiching among members of the six groups formed by employment status and gender is presented in Table 2, revealing that being sandwiched is slightly more common among women (10%) than among men (8%) and, particularly, more prevalent among employed women (13%) than among employed men (9%). While socially sandwiching of men and women in the paid labor force is attested among a distinct minority of the 50 + population of Europe, lifetime incidence throughout adulthood is likely to be higher.

## Results

The results of our multivariate fixed-effects analysis for women are shown in Table 3. They suggest that a transition into social sandwiching is associated with degradation of the psychological well-being of women in the paid labor force, manifested in increasing depressive symptoms and declining subjective well-being.[3] While estimated coefficients for these effects are relatively small (0.14 for Euro-D and − 0.25 for CASP12), we may reject the null hypothesis with a high level of confidence.

By contrast, we found that transitioning into social sandwiching was not associated with poorer psychological well-being among women who were either retired or otherwise out of the labor force. Thus, our results clearly suggest that it is specifically the triple challenge of devoting time and energy to paid work and providing support to younger and older family generations that takes a toll on women’s psychological well-being. Results reported in Table 3 also show that deteriorating physical health—measured by the number of chronic diseases and the GALI score—exacerbates depressive symptoms and worsens subjective well-being. The loss of a co-residing partner had a deleterious effect on women’s psychological well-being, whereas the birth of a grandchild was associated with an improvement of well-being for women who were not in paid work (either retired or otherwise not working). The last-mentioned finding, in the absence of a similar effect among working women, suggests that time constraints associated with paid work prevent working women from getting full pleasure from the birth of a grandchild.

The results of the analyses of the male subsample, shown in Table 4, differ from those observed among women. In line with previous research on the topic (Albertini et al. 2024), the regression results do not provide evidence that the transition into social sandwiching has a negative effect on working men’s psychological well-being. However, a negative and statistically significant decline in subjective well-being—an average decrease of about half a point on the CASP12 scale—is observed among retired men following their transition into social sandwiching. We also observe an association between the number of chronic diseases, GALI scores, and loss of a co-residing partner with an increase in the number of depression symptoms and a decrease in reported subjective well-being.

## Discussion

This study investigated the possibility of an association between becoming socially sandwiched while being in paid work and degradation of psychological well-being. Unlike most previous studies on the topic, we focused on the mature sandwich generation—the generation positioned between older parents and adult children (and possibly grandchildren). We also went beyond simply comparing the psychological well-being of sandwiched vs. not-sandwiched individuals by using a symmetric fixed-effects regression to estimate the consequences of a transition between these two conditions. The findings suggest a clear negative effect of becoming socially sandwiched among women in paid employment and no effect of such sandwiching among women not in employment. That these effects are consistently documented for both of our outcomes—number of depression symptoms and subjective well-being—strengthens our conclusion that the triple challenge of providing bi-directional intergenerational support while working produces detrimental emotional strain and reduces quality of life among middle-aged women. This finding corresponds with results reported in previous literature using cross-sectional analyses and provides support for the role-strain theory as a dynamic process. Despite the modest absolute size of the regression coefficients, it is worth noting that the observed effects of social sandwiching are multiplying through the growing population of women who defer retirement and have parents who survive to advanced old age. Indeed, we may expect the number of women who simultaneously provide care to frail parents and support adult children with young grandchildren while continuing to engage in productive employment up to and beyond typical retirement age to increase over the next several decades.

We also examined the same work and family dynamics among mature men. The results of these analyses are less conclusive than those observed for women. We found that the transition of employed men into social sandwiching is not statistically associated with change in psychological well-being. This suggests that employed men are better able to alleviate the strain associated with the transition to sandwiching either by attaining greater work flexibility or by engaging in support activities that are less emotionally and time consuming than those typical of women.

We found, however, that retired men experienced a decline in their perceived quality of life after transitioning into social sandwiching. Several social mechanisms may explain this effect, such as difficulty in transitioning from a breadwinner role to family-centric roles or dashing of expectations of engaging in leisure-oriented retirement. Nevertheless, our findings in reference to retired men and working women suggest that gender is a key factor to bear in mind when examining the consequences of social sandwiching among the older working-age population.

Beyond the specific gender patterns we observed, our findings underscore the value of donning transition-based analytic glasses. By tracking individuals as they move into social sandwiching, we provide evidence that psychological well-being is not merely associated with caregiving “status” but is sensitive to the dynamics of assuming new support and care responsibilities. This perspective helps reconcile the mixed evidence in earlier research literature that often relies on static comparisons that blur heterogeneity in the characteristics that predispose individuals to adopt multiple support roles. In addition, our inclusion of both women and men at mature working ages highlights gender differences in the deleterious effects of sandwiching on working women and retired men. Together, these innovations point toward the need for future research and policy to focus on life-course transitions and gender-specific vulnerabilities related to family and work roles.

This study has several limitations that deserve mention. First, we focused on multiple European countries but did not consider how cross-national variations in the generosity of their social policies may moderate the documented effects. Our decision to model transitions, together with the relatively low prevalence of social sandwiching, yielded sparse data in national contexts and prevented us from investigating how welfare regimes moderate the impact of work–family conflict. Although there are known differences among countries in the way social sandwiching impacts psychological well-being (Albertini et al. 2024), these differences are inconsequential in a fixed-effects context that allows only dynamic factors to be considered. Our inclusion of multiple European states, however, broadens and increases the relevance of our conclusions beyond previous investigations that tended to focus on one country only.

A second limitation relates to our treatment of labor-force participation as constant because dynamic treatment would require interacting it with sandwiching transitions, thus challenging the power of the model to detect meaningful effects. Almost two-thirds (64%) of respondents who changed their employment status did so by moving from work into retirement or another non-working category. Removing from consideration those who left work due to stress-inducing demands of social sandwiching may leave those remaining at work as a particularly robust set of individuals. This, however, represents a conservative bias that would countervail the hypothesis that being socially sandwiched while employed is psychologically disadvantageous.

A third limitation is that gender differences in caregiver stress may be driven by factors not considered here, such as the type of support provided, say, financial transfer as against personal care. That women shoulder more responsibility than men for time consuming and emotionally demanding personal care may explain the deleterious effects found among women. Further, gender differences in occupational status and type of employer may have implications for the level of stress imposed by work-related time demands. Jobs dominated by women tend to be of lower status and are less flexible in terms of taking time off for family care. Research showing that work factors contribute to sandwiched caregivers’ negative psychological well-being underscores the relevance of employer policies, such as caregiving leave and flextime, as mitigating factors (Hodgdon et al. 2023). The more generous employee benefits found in higher-status male-dominated occupations tend to attenuate caregiver stress (Grünwald et al. 2021). We suggest that future studies using larger data sets or population data from national registries may develop more nuanced models that would include the analysis of some of these factors with sufficient power.

We also point out that the conditions precipitating support may induce as much stress as the provision of support itself. Research shows that having an impaired family member is highly stressful even if they are not provided with care (Amirkhanyan and Wolf 2003). In addition, anticipated family events, such as impending financial demands of caring for a senescent parent or dealing with the birth of a grandchild, may induce older adults to continue to work to meet the pecuniary demands of care (Keene and Prokos 2007). Thus, stress may precede the behavioral response of caring for a loved one. These dynamics, we suggest, provide avenues for future research that are worth pursuing.

## Conclusions

In sum, this study expands the traditional focus of generational sandwiching—primarily centering on mothers caring for young children and older parents—to late middle-aged individuals who have adult children as well as very old parents at high risk of frailty. This rapidly growing segment of the population also increasingly participates in the paid labor force. Concomitantly, adult children of these middle-aged parents are in growing need of supplemental help with money, housing, and childcare.

Despite the limitations noted above, our results provide robust confirmation of the deleterious psychological impact on women of coupling social sandwiching with paid employment. The same disadvantage is not observed among men, reflecting known formal and informal workplace policies that favor men. Employers may address this issue by offering more flexible work arrangements and other accommodations that make work life more amenable to employees who attend to multiple family responsibilities; importantly, these work arrangements should be applied across a broad spectrum of occupations to ensure gender neutrality. In sum, our focus on social-sandwiching transitions among mature working-age women and men sets our findings within broader demographic trends of deferred retirement, population aging, and developmental delays of emerging adults—which together exacerbate the multiple demands that this middle generation faces. We hope our analysis points the way to future studies at the intersection of work, family, and gender, with the goal of developing policies that will narrow gender inequalities among those who juggle multiple family responsibilities while working until the age of pension eligibility.

## Figures and Tables

**Table 1:** Characteristics of the analytical sample

Employment status	*Female Respondents*	*Male Respondents*
	
Other, not in paid work	27.75	14.96
In paid work	48.53	59.62
Retired	23.71	25.42
% limitation with activities (GALI)	37.76	34.71
% has co-residing partner not paid work	63.87	63.86
% has co-residing partner in paid work	13.32	19.09
Average number of grandchildren	1.83	1.42
Average age	58.48	58.81
Number of respondents	26,604	20,169
Number of observations	69,849	51,260

**Table 2:** Total number of observations and percent socially sandwiched in each of the six separate subsamples

	*Female Respondents*		*Male Respondents*	
	Number of Observations	Percent Sandwiched	Number of Observations	Percent Sandwiched
*Other, not in paid*	19,386	8.65	7,670	5.16
*In paid work*	33,900	12.74	30,561	9.36
*Retired*	16,563	7.75	13,029	6.62
*Overall sample*	69,849	10.42	51,260	8.03

**Table 3:** Fixed effects by employment status, women

	Number of Depression Symptoms (Euro-D)			Subjective Well-being (CASP12)		
	*Other not in Paid Work*	*In Paid Work*	*Retired*	*Other not in Paid Work*	*In Paid Work*	*Retired*
Socially sandwiched	−0.0173	0.137***	0.0824	−0.0544	−0.246***	−0.0811
	(0.0764)	(0.0377)	(0.0845)	(0.171)	(0.0851)	(0.201)
Age (centered)	−0.0580***	−0.0232***	−0.0468***	0.0710***	0.0807***	0.0688***
	(0.00581)	(0.00363)	(0.00794)	(0.0130)	(0.00820)	(0.0189)
Number of chronic diseases	0.206***	0.170***	0.190***	−0.329***	−0.286***	−0.296***
	(0.0203)	(0.0149)	(0.0211)	(0.0455)	(0.0336)	(0.0503)
GALI	0.656***	0.374***	0.366***	−0.980***	−0.683***	−1.062***
	(0.0546)	(0.0309)	(0.0534)	(0.122)	(0.0699)	(0.127)
Number of grandchildren	0.0304*	0.00340	0.0494**	0.0510	0.00620	−0.0265
	(0.0166)	(0.0114)	(0.0221)	(0.0372)	(0.0257)	(0.0525)
Co-residing partner presence and employment status (REF: Yes, not in paid work)						
*No co-residing partner*	0.470***	0.340***	0.506***	−0.119	−0.404***	0.0994
	(0.125)	(0.0681)	(0.131)	(0.279)	(0.154)	(0.311)
*Yes co-residing partner in paid work*	−0.0399	0.0511	0.0333	0.121	_-_0.412***	0.163
	(0.117)	(0.0692)	(0.134)	(0.261)	(0.156)	(0.318)
Constant	2.226***	1.808***	1.888***	36.97***	40.00***	38.79***
	(0.0595)	(0.0352)	(0.0722)	(0.133)	(0.0794)	(0.172)
Observations	19,386	33,900	16,563	19,386	33,900	16,563

Note: Standard errors in parentheses;

*** p < 0.01,

** p < 0.05,

* p < 0.1

**Table 4:** Fixed effects by employment status, men

	Depression Symptoms (Euro-D)			Subjective well-bing (CASP12)		
	*Other not in Paid Work*	*In Paid Work*	*Retired*	*Other not in Paid Work*	*In Paid Work*	*Retired*
Socially sandwiched	0.0654	−0.0254	0.0725	−0.174	−0.150	−0.552**
	(0.181)	(0.0379)	(0.0894)	(0.444)	(0.101)	(0.235)
Age (centered)	−0.0569***	−0.0108***	−0.0251***	0.120***	0.0783***	0.0556***
	(0.0112)	(0.00321)	(0.00816)	(0.0274)	(0.00859)	(0.0214)
Number of chronic diseases	0.212***	0.139***	0.121***	−0.288***	−0.265***	_-_0.249***
	(0.0317)	(0.0135)	(0.0218)	(0.0779)	(0.0362)	(0.0573)
GALI	0.529***	0.331***	0.433***	−1.233***	−0.783***	−1.139***
	(0.0971)	(0.0290)	(0.0525)	(0.239)	(0.0775)	(0.138)
Number of grandchildren	0.0252	−0.00183	0.0316	0.0522	−0.0240	−0.00556
	(0.0297)	(0.0108)	(0.0220)	(0.0729)	(0.0290)	(0.0578)
Co-residing partner presence and employment status (REF: Yes, not in paid work)						
*No co-residing partner*	0.803***	0.265***	0.190	−1.158**	−0.281	−0.765*
	(0.211)	(0.0666)	(0.154)	(0.518)	(0.178)	(0.403)
*Yes, co-residing partner in paid work*	0.412*	−0.00723	0.0760	−0.319	0.216	0.207
	(0.215)	(0.0542)	(0.107)	(0.527)	(0.145)	(0.280)
Constant	1.751***	1.269***	1.371***	36.57***	39.98***	39.25***
	(0.116)	(0.0275)	(0.0625)	(0.285)	(0.0736)	(0.164)
Observations	7,670	30,561	13,029	7,670	30,561	13,029

Note: Standard errors in parentheses;

*** p < 0.01,

** p < 0.05,

* p < 0.1

## Data Availability

This paper uses data from SHARE Waves 1, 2, 4, 5, 6, 8 and 9 (DOIs: 10.6103/SHARE.w1.900, 10.6103/SHARE.w2.900, 10.6103/SHARE.w4.900, 10.6103/SHARE.w5.900, 10.6103/SHARE.w6.900, 10.6103/SHARE.w8.900, 10.6103/SHARE.w9.900) see Börsch-Supan et al. (2013) for methodological details. SHARE Data are available at the following URL: www.share-eric.eu

## Footnotes

[3] It is worth noting that the estimates are based on symmetric fixed effects. Given the limited statistical power of the data, it is not possible to fully estimate asymmetric effects and, thus, distinguish the effect of entering social sandwiching from that of exiting this status. Additional sensitivity analyses that treat social sandwiching as an absorbing state and, thus, censor observations to the right—that is, exclude them from the analytical sample when a respondent switches from socially sandwiched to not-socially sandwiched—yield similar results to those reported below.
